# Supplementary material for: Phytochrome-interacting factors directly suppress MIR156 expression to enhance shade-avoidance syndrome in Arabidopsis
Source: Nat Commun. 2017 Aug 24;8:348. doi: 10.1038/s41467-017-00404-y (PMC5570905; doi:10.1038/s41467-017-00404-y)
Supplement: Supplementary file 1 — Supplementary Information [file 41467_2017_404_MOESM1_ESM.pdf]

File name: Supplementary Information
Description: Supplementary Figures, Supplementary Tables and Supplementary References

File name: Supplementary Data 1
Description: Dataset for two-way ANOVA analysis

File name: Peer Review File
Description:

Supplementary Table 1. Primers Sequences Used in This Study

Primer ID	Primer Sequence (5'→3')	Purpose
JG01	ATTATGCCTCTCCCGAATTCATGCATCATTTTGTCCCTGAC	Cloning *PIF1* coding region into pJG4-5 vector for Y1H
JG02	TTCTCGAGTCGGCCGAATTCTTAACCTGTTGTGTGGTTTC	
JG03	ATTATGCCTCTCCCGAATTCATGCCTCTGTTTGAGCTTTTC	Cloning *PIF3* coding region into pJG4-5 vector for Y1H
JG04	TTCTCGAGTCGGCCGAATTCTCACGACGATCCACAAAACTG	
JG05	ATTATGCCTCTCCCGAATTCATGGAACACCAAGGTTGGAG	Cloning *PIF4* coding region into pJG4-5 vector *for* Y1H
JG06	TTCTCGAGTCGGCCGAATTCCTAGTGGTCCAAACGAGAACCG	
JG07	ATTATGCCTCTCCCGAATTCATGGAACAAGTGTTTGCTG	Cloning *PIF5* coding region into pJG4-5 vector for Y1H
JG08	TTCTCGAGTCGGCCGAATTCTCAGCCTATTTACCCATATG	
U01	ATTCGAGCTCGGTACCCGGG AGTGACAGATCCAACGGCAG	Cloning *MIR156A* promoter into pLacZi2μ vector for Y1H
U02	TCGACAGATCCCCGGGGTTTCTTTGCGTTTCTCTTGTC	
U03	ATTCGAGCTCGGTACCCGGG ATAAGTGCAGAGTCTAAGAC	Cloning *MIR156B* promoter into pLacZi2μ vector for Y1H
U04	TCGACAGATCCCCGGGGTTTTCTCTGTTGCATTCCTC	
U05	ATTCGAGCTCGGTACCCGGG CAGTGCCCTGAATGGATTACAC	Cloning *MIR156C* promoter into pLacZi2μ vector for Y1H
U06	TCGACAGATCCCCGGGGAGAGGAGAAGAGAGGAAG	
U07	ATTCGAGCTCGGTACCCGGG TAACTCTCTCTCTCTCTCTCTC	Cloning *MIR156D* promoter into pLacZi2μ vector for Y1H
U08	TCGACAGATCCCCGGGACTTCTTTTCCCCCATCAACA	
U09	ATTCGAGCTCGGTACCCGGG CTCAACATTTCCGGTTGACTAC	Cloning *MIR156E* promoter into pLacZi2μ vector for Y1H
U10	TCGACAGATCCCCGGGCCTCCTAATTACCTTTCACAC	
U11	ATTCGAGCTCGGTACCCGGG GACACATCCACAACCACCACTC	Cloning *MIR156F* promoter into pLacZi2μ vector for Y1H
U12	TCGACAGATCCCCGGGCCATCAATTCCTCACCACTC	
U13	ATTCGAGCTCGGTACCCGGG CTGTGAAGTACATAATGGCTG	Cloning *MIR156G* promoter into pLacZi2μ vector for Y1H
U14	TCGACAGATCCCCGGGTGCTATTCCCTTACCTCCTTTAG	
U15	ATTCGAGCTCGGTACCCGGG ATCTGCTTCTTCAGTCAATTC	Cloning *MIR156H* promoter into pLacZi2μ vector for Y1H
U16	TCGACAGATCCCCGGGCTTCTTCTCGGGAGGAATAGAAG	
U17	CGAGCTCGGTACCCGGGGAACCATGCATCTGATTTACAG	Cloning *SPL2* promoter into pLacZi2μ vector for Y1H
U18	GTCGACAGATCCCCGGGGGTTGCATATTAAAGTGTCTG	
U19	CGAGCTCGGTACCCGGGTATTCAATTAACGTTGCTCG	Cloning *SPL3* promoter into pLacZi2μ vector for Y1H
U20	GTCGACAGATCCCCGGGGAGAAACTGAGAAACACTGTG	
U21	CGAGCTCGGTACCCGGGACCCATAGGTTATGTAAGTTAC	Cloning *SPL4* promoter into pLacZi2μ vector for Y1H
U22	GTCGACAGATCCCCGGGAACTTAGGATCTGATCACTG	
U23	CGAGCTCGGTACCCGGGGATTCTTTCTTTAGCTGGTC	Cloning *SPL5* promoter into pLacZi2μ vector for Y1H
U24	GTCGACAGATCCCCGGGGTGACATCCTTTGTCGATGTAG	
U25	CGAGCTCGGTACCCGGGGGTATAGTTGGATGTTCTATGGC	Cloning *SPL9* promoter into pLacZi2μ vector for Y1H
U26	GTCGACAGATCCCCGGGGGTTCAAGTTCAACGTACACG	
U27	CGAGCTCGGTACCCGGGCATGGCGTTCTGAAACTCACTC	Cloning *SPL15* promoter into pLacZi2 vector for Y1H
U28	GTCGACAGATCCCCGGGGACAGAGAAAGAGATGCTTC	
AD1	GGAGGCCAGTGAATTCATGCATCATTTTGTCCCTGAC	Cloning *PIF1* coding region into pGADT7 vector for Y2H
AD2	CACCCGGGTGGAATTCTTAACCTGTTGTGTGGTTTC	
AD3	GGAGGCCAGTGAATTCATGCCTCTGTTTGAGCTTTTC	Cloning *PIF3* coding region into

AD4	CACCCGGGTGGAATTCTCACGACGATCCACAAAACTG	pGADT7 vector for Y2H
AD5	GGAGGCCAGTGAATTCATGGAACACCAAGGTTGGAG	Cloning *PIF4* coding region into pGADT7 vector for Y2H
AD6	CACCCGGGTGGAATTCCTAGTGGTCCAAACGAGAACCG	
AD7	GGAGGCCAGTGAATTCATGGAACAAGTGTTTGCTG	Cloning *PIF5* coding region into pGADT7 vector for Y2H
AD8	CACCCGGGTGGAATTCTCAGCCTATTTTACCCATATG	
BK1	CATGGAGGCCGAATTCATGGAGTGTAATGCAAAGC	Cloning *SPL2* coding region into pGBKT7 vector for Y2H
BK2	GGATCCCCGGGAATTCTCAGTTATAAAACTGGTTCA	
BK3	CATGGAGGCCGAATTCATGAGTATGAGAAGAAGCAAAG	Cloning *SPL3* coding region into pGBKT7 vector for Y2H
BK4	GGATCCCCGGGAATTCTTAGTCAGTTGTGCTTTTC	
BK5	CATGGAGGCCGAATTCATGGAGGGTAAGAGATCAC	Cloning *SPL4* coding region into pGBKT7 vector for Y2H
BK6	GGATCCCCGGGAATTCCTATCTAATCTGTGGTCGC	
BK7	CATGGAGGCCGAATTCATGGAGGGTCAGAGAACAC	Cloning *SPL5* coding region into pGBKT7 vector for Y2H
BK8	GGATCCCCGGGAATTCTTATCTGATCTGTGGTCGCTTG	
BK9	CATGGAGGCCGAATTCATGGAGATGGGTTCCAACTC	Cloning *SPL9* coding region into pGBKT7 vector for Y2H
BK10	GGATCCCCGGGAATTCTCAGAGAGACCAGTTGGTATG	
BK11	CATGGAGGCCGAATTCATGGACTGCAACATGGTATC	Cloning *SPL10* coding region into pGBKT7 vector for Y2H
BK12	GGATCCCCGGGAATTCTCAGATGAAATGACTAGGG	
BK13	CATGGAGGCCGAATTCATGGACTGCAACATGGTATC	Cloning *SPL11* coding region into pGBKT7 vector for Y2H
BK14	GGATCCCCGGGAATTCCTATTTGGTACAACATCAT	
BK15	CATGGAGGCCGAATTCATGGAGTTGTTAATGTGTTC	Cloning *SPL15* coding region into pGBKT7 vector for Y2H
BK16	GGATCCCCGGGAATTCTCAAAGAGACCAATTGAAATG	
Mut01	ACATGATGTTTTGTCAAAAAAGGGTTTAGAAGATTTCTAAGGTC ATCTTACAGTGAGTGGGTGTTTAATTAACTTCATTAAAGT	Mutagenesis of the G-box of *MIR156B* promoter
Mut02	ACTTTAATGAAGTTAATTAAACACCCACTCACTGTAAGATGACC TTAGAAATCTTCTAAACCCTTTTTTGACAAAACATCATGT	
Mut03	AATATTAATTTTTCTAATTAAACTCTTTCAAGTGAAACTAATAAC AGGTCATTGCATGAAAATTCTTTTCAACGTCCGTTTTCGTTTCTA AAACGC	Mutagenesis of the G-box of *MIR156D* promoter
Mut04	GCGTTTTAGAAACGAAAACGGACGTTGAAAAGAATTTTCATGC AATGACCTGTTATTAGTTTCACTTGAAAGAGTTTAATTAGAAAA ATTAATATT	
Mut05	CGTGGTCAGCCCCTGAGGAGAAGGTCAGTGGGAACGAATATGC CGCCC	Mutagenesis of the first G-box of *MIR156E* promoter
Mut06	GGGCGGCATATTCGTTCCCACTGACCTTCTCCTCAGGGGCTGAC CACG	
Mut07	CTTTCCACTTTCGGCGTGGGAATAGGTCAGTCAGCCCCTGAGG AGACACGTG	Mutagenesis of the second G-box of *MIR156E* promoter
Mut08	CACGTGTCTCCTCAGGGGCTGACTGACCTATTCCCACGCCGAA AGTGGAAAG	
Mut09	GAGAATGCCCAGCCTGTAGGTCATCGTAATGGGGTTTGTTCGTC GTCCAACCCTA	Mutagenesis of the G-box of *MIR156F* promoter
Mut10	TAGGGTTGGACGACGAACAAACCCCATTACGATGACCTACAGG	

	CTGGGCATTCTC	
Mut11	TACAGGGCCCACTTTTTATTTCGTCAATAGGAGGTCATTGATTTAGATTGACACGAGTCGAGTATATAGATAG	Mutagenesis of the G-box of *MIR156H* promoter
Mut12	CTATCTATATACTCGACTCGTGTCAATCTAAATCAATGACCTCCTATTGACGAAATAAAAAGTGGGCCCTGTA	
GUS01	TTGCATGCCTGCAGGTCGACATAAGTGCAGAGTCTAAGAC	Cloning *MIR156B* promoter into pBI101 vector
GUS02	GGGGATCCTCTAGAGTCGACGTTTTCTCTGTTGCATTCCTC	
GUS03	TTGCATGCCTGCAGGTCGACTAACTCTCTCTCTCTCTCTC	Cloning *MIR156D* promoter into pBI101 vector
GUS04	GGGGATCCTCTAGAGTCGACACTTCTTTTCCCCCATCAACA	
GUS05	TTGCATGCCTGCAGGTCGACCTCAACATTTCCGGTTGACTAC	Cloning *MIR156E* promoter into pBI101 vector
GUS06	GGGGATCCTCTAGAGTCGACCCTCCTAATTACCTTTCACAC	
GUS07	TTGCATGCCTGCAGGTCGACGACACATCCACAACCACCACTC	Cloning *MIR156F* promoter into pBI101 vector
GUS08	GGGGATCCTCTAGAGTCGACCCATCAATTCCTCACCACTC	
GUS09	TTGCATGCCTGCAGGTCGACATCTGCTTCTTCAGTCAATTC	Cloning *MIR156H* promoter into pBI101 vector
GUS10	GGGGATCCTCTAGAGTCGACCTTCTTCTCGGGAGGAATAGAAG	
OE01	GATCCCCGGGTACCGAGCTCAGAAGAGGGAGAGATGGTGA	Cloning *MIR156B* fragment into pCPB1-1 vector for overexpression
OE02	GATCGGGGAAATTCGAGCTCTCAAGCAGGCAGAGATAGG	
OE03	GATCCCCGGGTACCGAGCTCCATCGGTTTCTGGACTAATTG	Cloning *MIR156D* fragment into pCPB1-1 vector for overexpression
OE04	GATCGGGGAAATTCGAGCTCCATAACTAGAACAATGGAATAAG	
OE05	GATCCCCGGGTACCGAGCTCCAAAGATAGAAAGATGTAAGGTC	Cloning *MIR156E* fragment into pCPB1-1 vector for overexpression
OE06	GATCGGGGAAATTCGAGCTCGCTTCATCGTACGTTATAGATC	
OE07	GATCCCCGGGTACCGAGCTCGGTATCCGTATATCTCTATAT	Cloning *MIR156F* fragment into pCPB1-1 vector for overexpression
OE08	GATCGGGGAAATTCGAGCTCCTAAATGAAATGCTGTAGAGAG	
OE09	GATCCCCGGGTACCGAGCTCGAAGAGGTAAGAAAGTGAAAGGGA	Cloning *MIR156H* fragment into pCPB1-1 vector for overexpression
OE10	GATCGGGGAAATTCGAGCTCTCTCAGAATCTTGAACAAAAGC	
Q01	GTTAAAACTCAGATCTAACACAAAG	qPCR of primary *MIR156A*
Q02	GAGAACGAAGACAGGCCAAAG	
Q03	AGAGGGAGAGATGGTGATTGAGGAATG	qPCR of primary *MIR156B*
Q04	GAGAGGTCAAGCAGGCAGAGATAGG	
Q05	ACTCCAACACCTTCAAAGTCTGC	qPCR of primary *MIR156C*
Q06	GAGAGAGAAAGTGAGAGATGGGAAC	
Q07	CAGAAGAGAGTGAGCACACAAAGGG	qPCR of primary *MIR156D*
Q08	GTGAGCACGCAAAAGCAACCATATAC	
Q09	GGTCTAGAGTCTTGTTCTTAATCCCC	qPCR of primary *MIR156E*
Q10	CCTAATTACCTTTCACACTCTACGC	
Q11	TGGTGAGGAATTGATGGTGACA	qPCR of primary *MIR156F*
Q12	CCTTCAAATATGCAAGAAAGCCAC	
Q13	GGGACCGAGGCTAATATAACATCAA	qPCR of primary *MIR156G*
Q14	ATGCTAGAAAAAGAGCCATGTGTGC	
Q15	AGCACAACCTGGGATTAGCAAA	qPCR of primary *MIR156H*

Q16	AACATACGCTCATGACACGATCA	
Q17	CGCTGACAGAAGAGAGTGAGCAC	qPCR of mature *MIR156*
Q18	AGATTTCCGATACCGAGCACA	qPCR of *SPL2*
Q19	TTGGAGGTTGCTTGAGGGATG	
Q20	ACCGCGGATATGAGCAAAGCCA	qPCR of *SPL3*
Q21	GAGCGCGTGAAACCTGCTGC	
Q22	TGGAGAAGGATCAGGTCGGAGAGG	qPCR of *SPL4*
Q23	CAGAGTGACCGTGGCTTTTGGT	
Q24	GGTCAGAGAACACAACGCCGGG	qPCR of *SPL5*
Q25	CCTGGCACAGTCGCGATGGA	
Q26	CCACGGAAGTATCCTCCATTT	qPCR of *SPL6*
Q27	TATTCCTGCTTCACATCACCA	
Q28	CAGGCAGACTGTTCACCAGA	qPCR of *SPL7*
Q29	AGTTTGACGGGACCTGAATG	
Q30	CAAGGTTCAGTTGGTGGAGGA	qPCR of *SPL9*
Q31	TGAAGAAGCTCGCCATGTATTG	
Q32	AGCACCCTCTCTTTCTCTGCGT	qPCR of *SPL10*
Q33	CGGCCACGGGAGTGTGTTTGAT	
Q34	CACTTATGATACAAAGCCTAGACAA	qPCR of *SPL11*
Q35	GGGGATCCGAAGAGGTTGACA	
Q36	GGGAAATAGTCTTGTAAGCGTTGC	qPCR of *SPL13*
Q37	TGGGACAAAGAAAGTGGTGGT	
Q38	GTGGTCAACCGCAAGATCAGT	qPCR of *SPL15*
Q39	TGAGCCATTGTAACCTTATCG	
Q40	TATCGGATGACGATTCTTCGTGCAG	qPCR of *PP2A*
Q41	GCTTGGTCGACTATCGGAATGAGAG	
Q42	AACACCCACTCACTGTAAG	ChIP-PCR of *MIR156B* fragment
Q43	GAGGATGGTAGATAGGAGC	
Q44	TAGGGTTTTGGAGAGATCTG	ChIP-PCR of *MIR156B* fragment
Q45	GCCAAATTTGAGAGAGAGAG	
Q46	CTTCCTTATTCCATTGTTC	ChIP-PCR of *MIR156D* fragment
Q47	TTGTGTTCTTATCTGTCTC	
Q48	ATCTGTACTTGTGTCATG	ChIP-PCR of *MIR156D* fragment
Q49	GAGAGTGAGCACGCAAAAGC	
Q50	ATGAGATGACCCACTTGAC	ChIP-PCR of *MIR156E* fragment
Q51	TGACCACAGACAGAACAAG	
Q52	CGACAGGTCTCAGTTTCTTC	ChIP-PCR of *MIR156E* fragment
Q53	GTGACAGAGAGAGAGTAAGC	
Q54	AACACAAACCGAAACCCAC	ChIP-PCR of *MIR156F* fragment
Q55	ATCTTGTGATGGTTATGGG	
Q56	AGGTATCCGTATATCTCTA	ChIP-PCR of *MIR156F* fragment
Q57	GAGAAGGGGGTGACGGATAG	
Q58	TTGCTCTCTCTCGCCACAAG	ChIP-PCR of *MIR156H* fragment

Q59	GTCGGAAGTTGCTTTCACG	
Q60	CTATCACACACTATAGCCG	ChIP-PCR of *MIR156H* fragment
Q61	CGCAATGATGGTGGCAGAAG	
Q62	CAACGAACAAATCACAGAAAACATG	ChIP-PCR of *PP2A*
Q63	AAAGGTAAAGAAGACAGCAACGAATT	
P01	TGGATCCCCGGAATTCGAAGTTCATAATCTCTCAGAAAGG	Cloning *PIF5* bHLH domain into pGEX-4T-1 vector
P02	GTCGACCCGGGAATTCTCATTGGAGTTGCATTTGAAGTGAT	
S01	GATCCATCGATAGTACTGTCGACATGCATCATTTGTCCCTGAC	Cloning *PIF1* coding region into pSPYCE vector
S02	GGAGCGGTACCCTCGAGGTCGACTTAACCTGTTGTGTGGTTTC	
S03	GATCCATCGATAGTACTGTCGACATGCCTCTGTTTGAGCTTTTC	Cloning *PIF3* coding region into pSPYCE vector
S04	GGAGCGGTACCCTCGAGGTCGACTCACGACGATCCACAAAACTG	
S05	GATCCATCGATAGTACTGTCGACATGGAACACCAAGGTTGGAG	Cloning *PIF4* coding region into pSPYCE vector
S06	GGAGCGGTACCCTCGAGGTCGACCTAGTGGTCCAAACGAGAACCG	
S07	GATCCATCGATAGTACTGTCGACATGGAACAAGTGTTTGCTG	Cloning *PIF5* coding region into pSPYCE vector
S08	GGAGCGGTACCCTCGAGGTCGACTCAGCCTATTTACCCATATG	
GR01	GAGATCGAATTCCCATGGATAAGTGCAGAGTCTAAGAC	Cloning *MIR156B* promoter into pGREEN 0800 vector
GR02	TTTTGGCGTCTTCCATGGTTTTCTCTGTTGCATTCCTC	
GR03	GAGATCGAATTCCCATGGATGCGTGTGTGTTTGTGTC	Cloning *MIR156D* promoter into pGREEN 0800 vector
GR04	TTTTGGCGTCTTCCATGGCACAAGTACAGATCGAAGG	
GR05	GAGATCGAATTCCCATGGCTCAACATTTCCGGTTGAC	Cloning *MIR156E* promoter into pGREEN 0800 vector
GR06	TTTTGGCGTCTTCCATGGCCTCCTAATTACCTTTCAC	
GR07	GAGATCGAATTCCCATGGGACACATCCACAACCACCACTC	Cloning *MIR156F* promoter into pGREEN 0800 vector
GR08	TTTTGGCGTCTTCCATGGCCATCAATTCCTCACCACTC	
GR09	GAGATCGAATTCCCATGGATCTGCTTCTTCAGTCAATTC	Cloning *MIR156H* promoter into pGREEN 0800 vector
GR10	TTTTGGCGTCTTCCATGGCTTCTTCTCGGGAGGAATAG	
E01	CATATTCGTTCCCACCACGTGTCTCCTCAGGGGCTGACCACGTGATTCCCACGCCGAAAG	Probe for MIR156E EMSA
E02	CTTTCGGCGTGGGAATCACGTGGTCAGCCCCTGAGGAGACACGTGGTGGGAACGAATATG	
E03	CATATTCGTTCCCACTGACCTTCTCCTCAGGGGCTGACTGACCTATTCCCACGCCGAAAG	Mutant probe for MIR156E EMSA
E04	CTTTCGGCGTGGGAATAGGTCAGTCAGCCCCTGAGGAGAAGGTCAGTGGGAACGAATATG	
E05	AACAAACCCCATTACGACACGTGACAGGCTGGGCATTCTCCACATGCTTTCTTGCCGAAA	Probe for MIR156F EMSA
E06	TTTCGGCAAGAAAGCATGTGGAGAATGCCCAGCCTGTCACGTGTCGTAATGGGGTTTGTT	
E07	AACAAACCCCATTACGATGACCTACAGGCTGGGCATTCTCCACATGCTTTCTTGCCGAAA	Mutant probe for MIR156F EMSA
E08	TTTCGGCAAGAAAGCATGTGGAGAATGCCCAGCCTGTTGACCTTCGTAATGGGGTTTGTT	

Supplementary Table 2. The number of G-box or PBE in putative *SPL* promoters

	Length	G-box (5′ CACGTG3′)	PBE (5′ CACATG3′)
pSPL2	3257 bp	1	2
pSPL3	2917 bp	1	2
pSPL4	3077 bp	1	2
pSPL5	2744 bp	1	0
pSPL9	3021 bp	0	5
pSPL10	1747 bp	0	0
pSPL11	2738 bp	0	0
pSPL15	1252 bp	0	1

Supplementary Figure 1

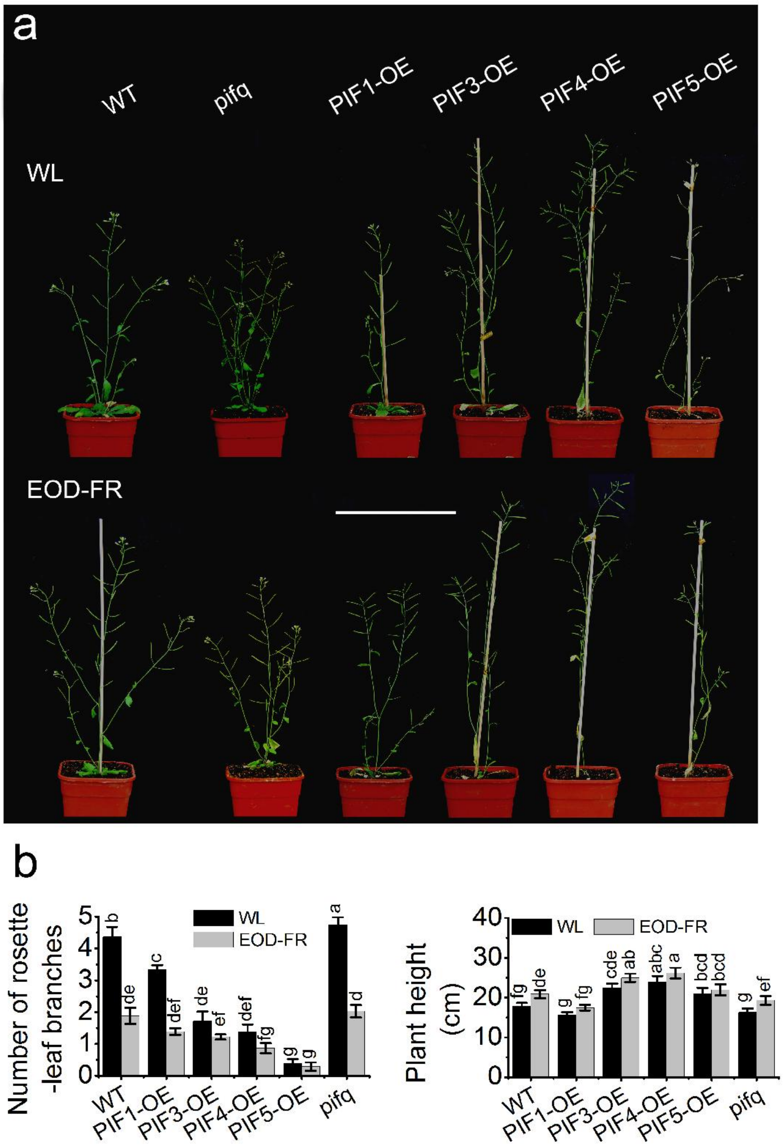

Supplementary Figure 1. Phenotypic analysis of adult *PIF* overexpessors in response to simulated shade

(a) The plant height and rosette-leaf branches between the adult *PIF* overexpessors, *pifq* and wild type plants grown under normal high R:FR (WL) conditions and simulated shade (EOD-FR) conditions. Eight-day-old seedlings grown under WL were transferred into the soil under WL conditions with or without EOD-FR treatment for four weeks before phenotypic analysis. Bar=10 cm. (b) Quantification of plant height and rosette-leaf branches of the *PIF* ovexpressors, *pifq* and wild type. Values given are means ± SD (n=12). Different letters indicate significant differences by two-way ANOVA.

Supplementary Figure 2

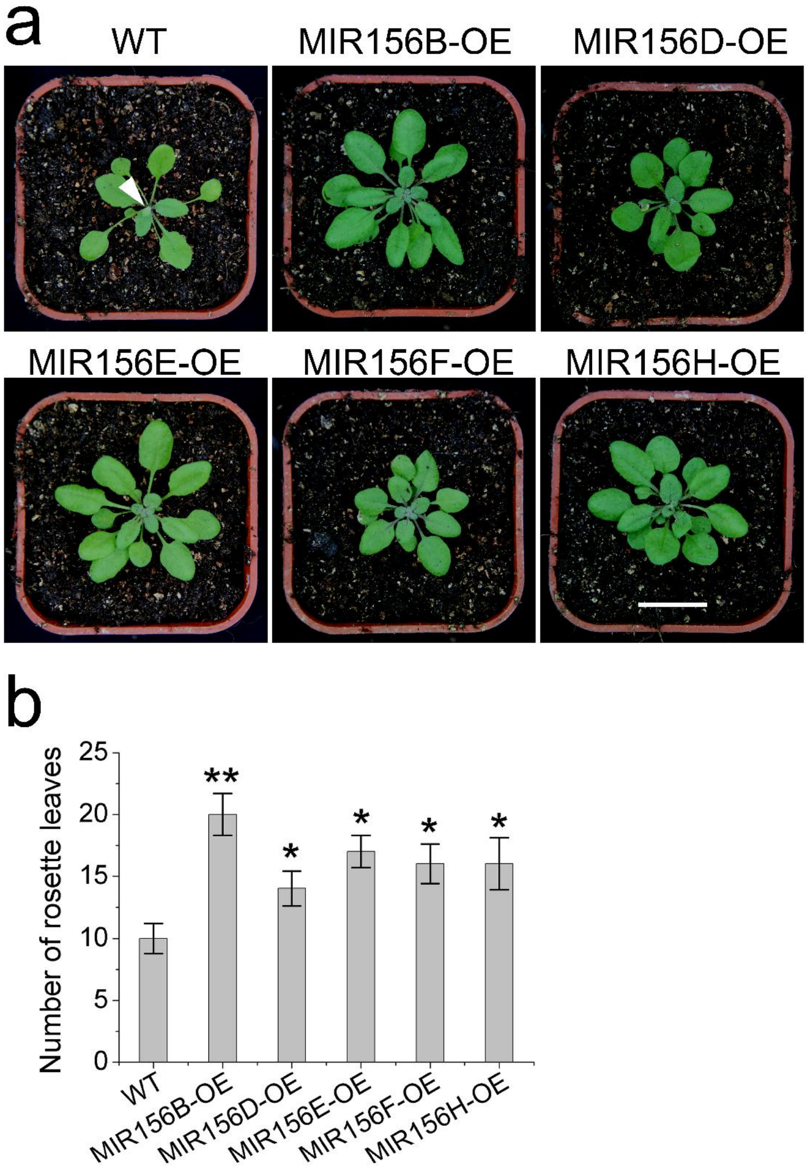

Supplementary Figure 2. Overexpressors of *MIR156* members show similar phenotypes

Overexpressors of *MIR156B*, *MIR156D*, *MIR156E*, *MIR156F* and *MIR156H* shows more rosette leaves, prolonged expression of juvenile vegetative traits and late flowering compared with wild type. Values given are means ± SD (n=12). *P<0.05 and **P<0.01 by a Student's *t* -test. Arrow indicates the bolting. Bar=2 cm.

Supplementary Figure 3

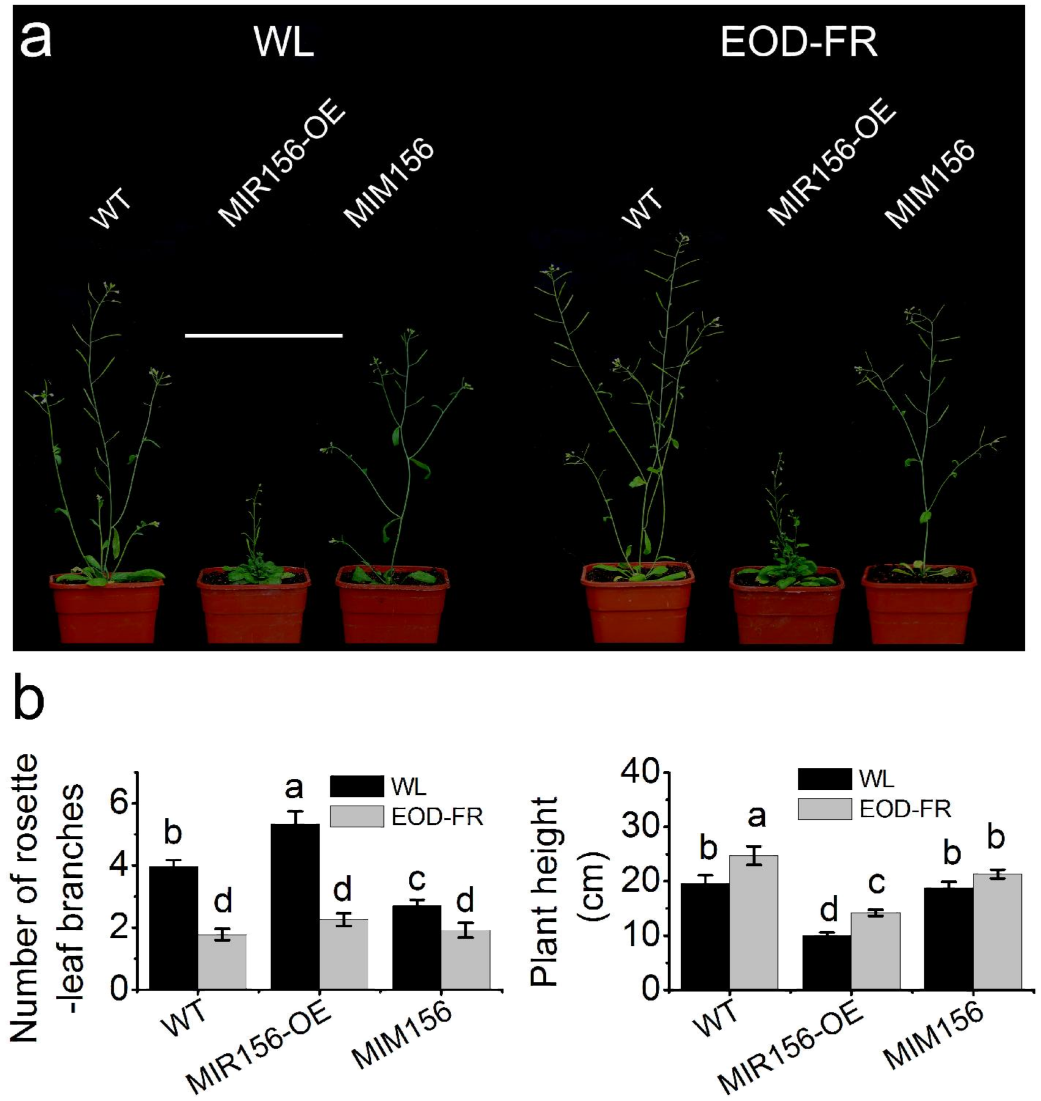

Supplementary Figure 3. Phenotypic analysis of *MIR156* overexpressor in response to simulated shade

(a) The plant height and rosette-leaf branches of *MIR156* overexpessor and wild type plants grown under high R:FR (WL) conditions or treated with EOD-FR. Eight-day-old seedlings grown under WL were transferred into the soil under WL conditions with or without EOD-FR treatment for four weeks before phenotypic examination. Bar=10 cm. (b) Quantification of plant height and rosette-leaf branches of *MIR156* overexpessor and wild type plants. Values given are means ± SD (n=12). Different letters indicate significant differences by two-way ANOVA.

Supplementary Figure 4

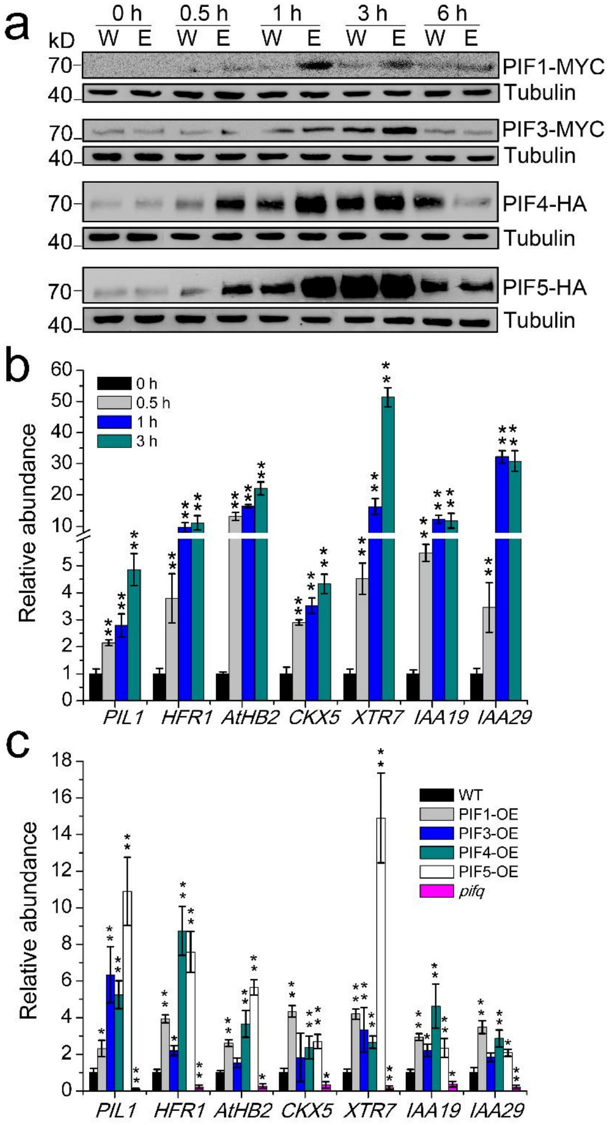

Supplementary Figure 4. Increased accumulation of PIF proteins and up-regulation of representative shade-avoidance marker genes in response to EOD-FR treatment

(a) PIF protein accumulation in the PIF-OE lines rapidly increases when exposed to EOD-FR treatment. Two-week-old seedlings over-expressing MYC or HA -tagged PIF proteins (PIF1-MYC, PIF3-MYC, PIF4-HA and PIF5-HA) grown under normal high R:FR conditions were exposed to EOD-FR for 15 minutes and harvested at the given time points before immunoblot analysis. Tubulin was used as the internal control. W: white light; E: EOD-FR. (b) qRT-PCR showing that expression of shade-avoidance marker genes are up-regulated in response to the EOD-FR treatment.

Eight-day-old wild type seedling grown under high R:FR were treated with 15-minute EOD-FR and harvested at the given time points for RNA extraction. Values given are means ± SD (n=3). *P<0.05 and **P<0.01 by a Student's *t* -test. (c) PIFs up-regulate the expression of shade-avoidance marker genes. The expression was determined by quantitative RT-PCR in 8-day-old seedlings grown under high R:FR conditions. *PP2A* was used as reference gene. Values given are means ± SD (n=3). *P<0.05 and **P<0.01 by a Student's *t* -test.

Supplementary Figure 5

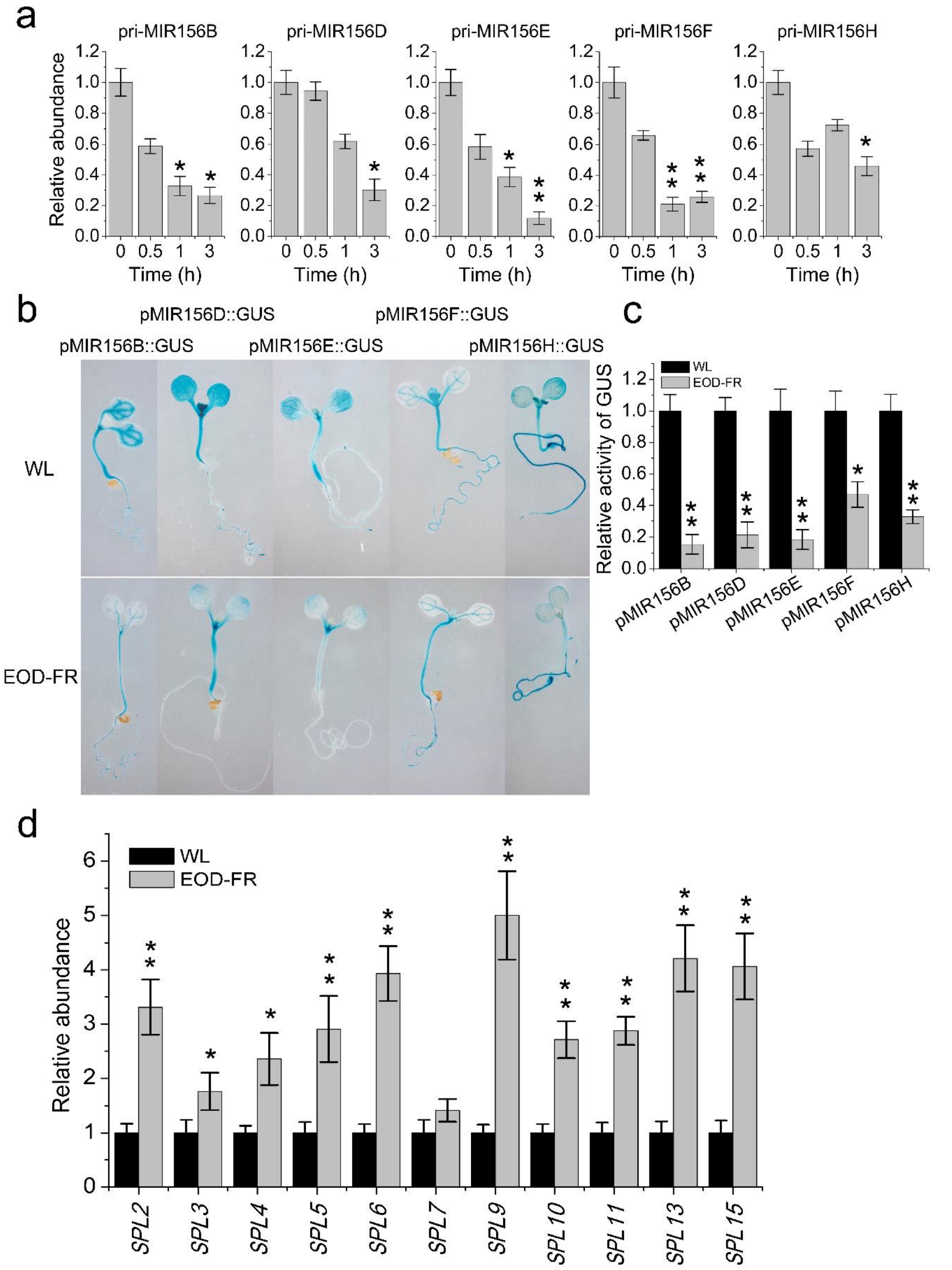

Supplementary Figure 5. The primary transcript level of *MIR156* declines while several *MIR156* targeted *SPL*s increase when exposed to EOD-FR treatment

(a) The relative expression levels of individual primary *MIR156* in 2-week-old wild type seedlings grown under WL or treated with EOD-FR. Values given are means ± SD (n=3). *P<0.05 and **P<0.01 by a Student's *t* -test. (b) GUS staining of 8-day-old *pMIR156::GUS* seedlings grown under high R:FR conditions or treated with EOD-FR.

EOD-FR treatment led to a significant reduction of GUS staining in the seedlings. Bar=200 µm. (c) Fluorometric quantification of GUS activity. Relative activity is normalized to the protein content in the plant extracts. Activities are plotted as percent of activity in the controls (WL). Homozygous T3 transgenic plants were used in the assay. Values given are means ± SD (n=3). *P<0.05 and **P<0.01 by a Student's *t* -test. (d) Comparison of *SPL* genes expression levels between WL and EOD-FR conditions. Eight-day-old wild type seedlings grown under WL conditions were treated with or without EOD-FR for 6 days and then harvested for RNA extraction and quantitative RT-PCR analyses. Values given are means ± SD (n=3). *P<0.05 and **P<0.01 by a Student's *t* -test.

Supplementary Figure 6

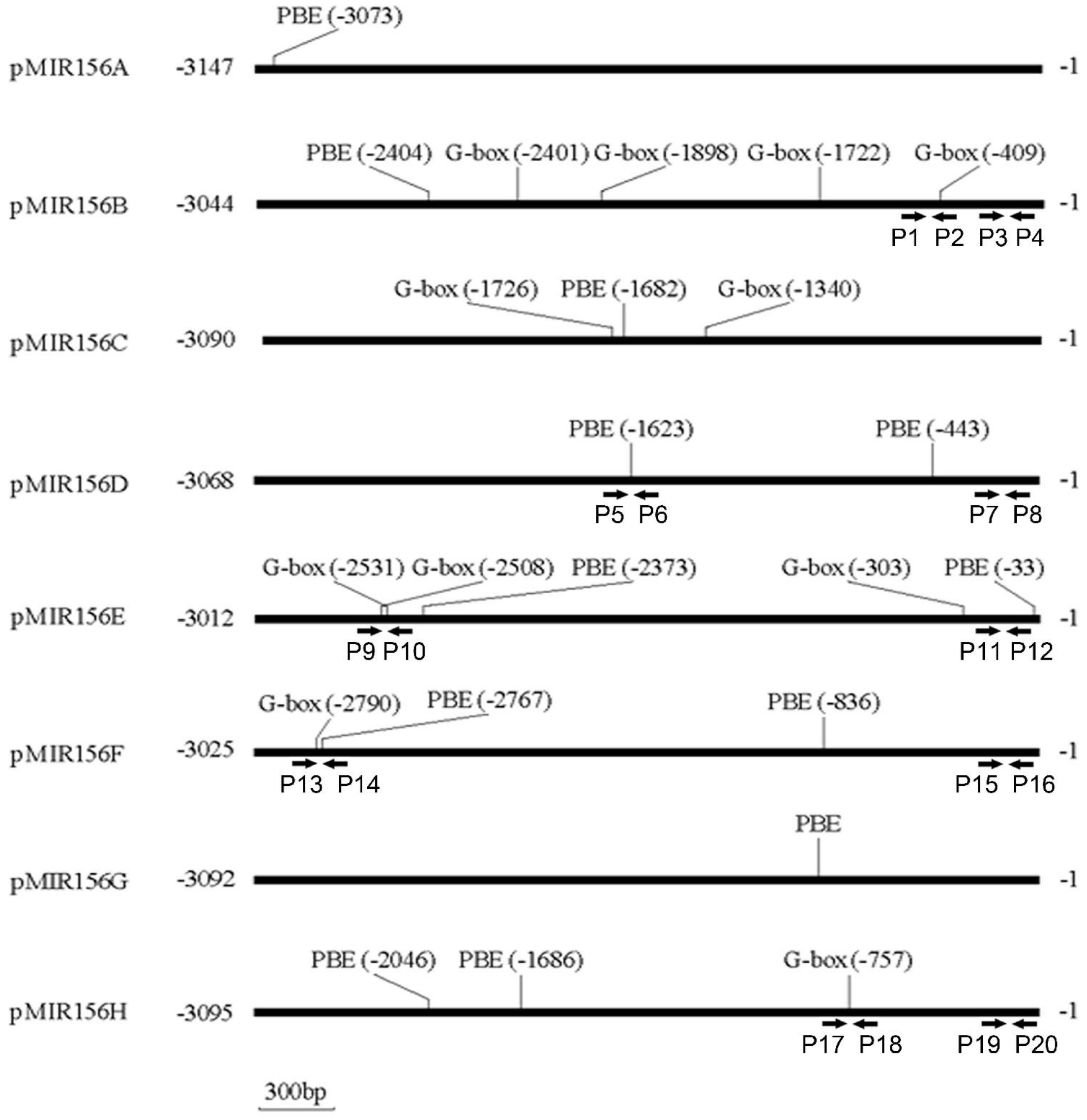

Supplementary Figure 6. All putative *MIR156* promoters harbor the putative PIF binding elements (G-box or PBE-box)

Around 3-kb upstream promoter regions of each *MIR156* gene were obtained from TAIR (The Arabidopsis Information Resource, www.arabidopsis.org) to search for the PIF binding site G-box (5′-CACGTG-3′) or PBE-box (5′-CACATG-3′). The position of the first base of each mature *miR156* was designed +1 in this study. The arrow pairs indicate the fragments amplified by ChIP-PCR in this study.

Supplementary Figure 7

a

```
pMIR156B    -428   acacccactcactgtaagaCACGTGtagaaatcttctaaaccctt -383
pMIR156Bm   -428   acacccactcactgtaagaTGACCTtagaaatcttctaaaccctt -383

pMIR156D    -1622  gaaaagaattttcatgcaaCACATGgttattagtttcacttgaaa -1577
pMIR156Dm   -1622  gaaaagaattttcatgcaaTGACCTgttattagtttcacttgaaa -1577

pMIR156E    -1542  ttcgttcccacCACGTGtctcctcaggggctgacCACGTGattcc -2497
pMIR156Em   -1542  ttcgttcccacTGACCTtctcctcaggggctgacTGACCTattcc -2497

pMIR156F    -2809  cgaacaaaccccattacgaCACGTGacaggctgggcattctccac -2764
pMIR156Fm   -2809  cgaacaaaccccattacgaTGACCTacaggctgggcattctccac -2764

pMIR156H    -776   ctcgtgtcaatctaaatcaaCACGTGcctattgacgaaataaaaa -731
pMIR156Hm   -776   ctcgtgtcaatctaaatcaaTGACCTcctattgacgaaataaaaa -731
```

b

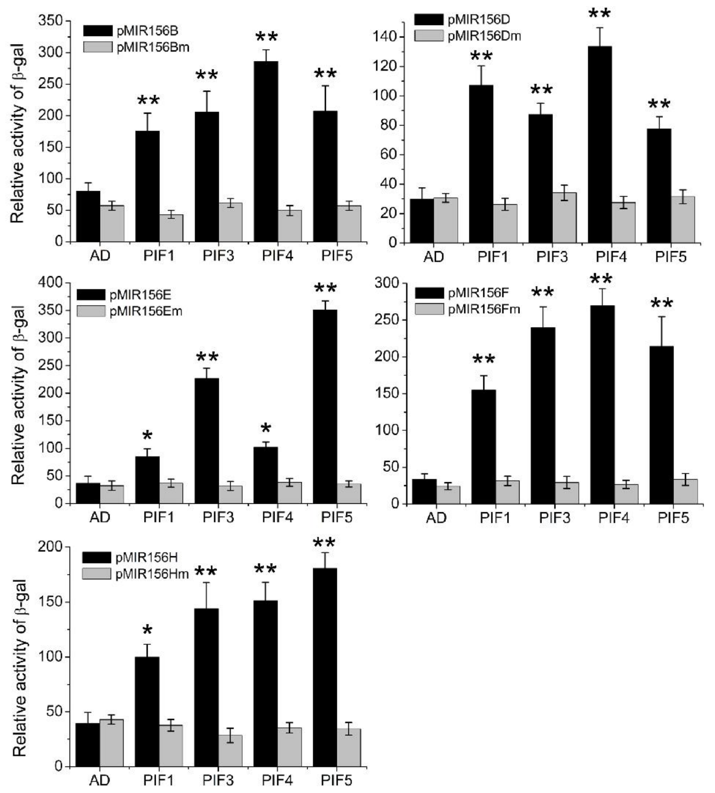

Supplementary Figure 7. Mutagenesis of the G-box in the *MIR156* promoters and quantification of PIF binding activity.

(a) Mutagenesis of the G-box in the *MIR156* promoters. The G-box (5′-CACGTG-3′) or PBE-box (5′-CACATG-3′) was mutated into 5′-TGACCT-3′ in this study. (b) Quantification of activity of PIF binding to *MIR156* prompters. At least 6 independent yeast clones were used for activity determination. Values given are means ± SD ($n \geq 6$). *$P<0.05$ and **$P<0.01$ by a Student's *t* -test.

Supplementary Figure 8

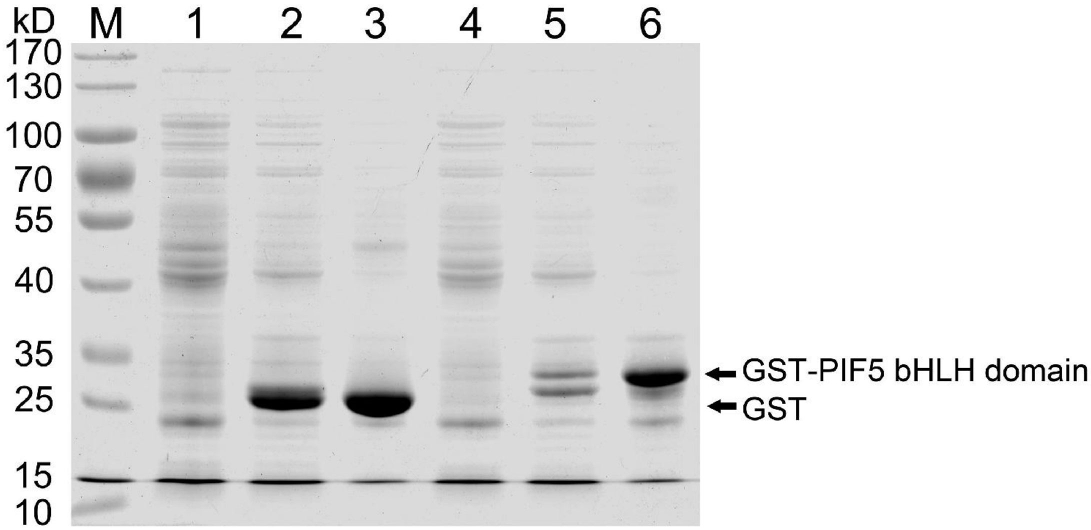

Supplementary Figure 8. SDS-PAGE analysis of purified GST and recombinant GST-PIF5 bHLH domain proteins.

The truncated fragment of *PIF5* cDNA encoding the bHLH domain (52 aa, Glu_{259} -Gln_{310}) was PCR amplified and cloned into the vector GEX-4T-1 to generate pGEX-4T-PIF5 construct for GST-PIF5 bHLH domain fusion protein. M: molecular mass standard; Line 1 and 2: *Escherichia coli* BL21 (DE3) harboring GST without or with induction by IPTG, respectively; Line 3: purified GST proteins; Line 4-5: *Escherichia coli* BL21 (DE3) harboring recombinant GST-PIF5 bHLH domain without or with induction by IPTG, respectively; Line 6: purified recombinant GST-PIF5 bHLH domain proteins.

Supplementary Figure 9

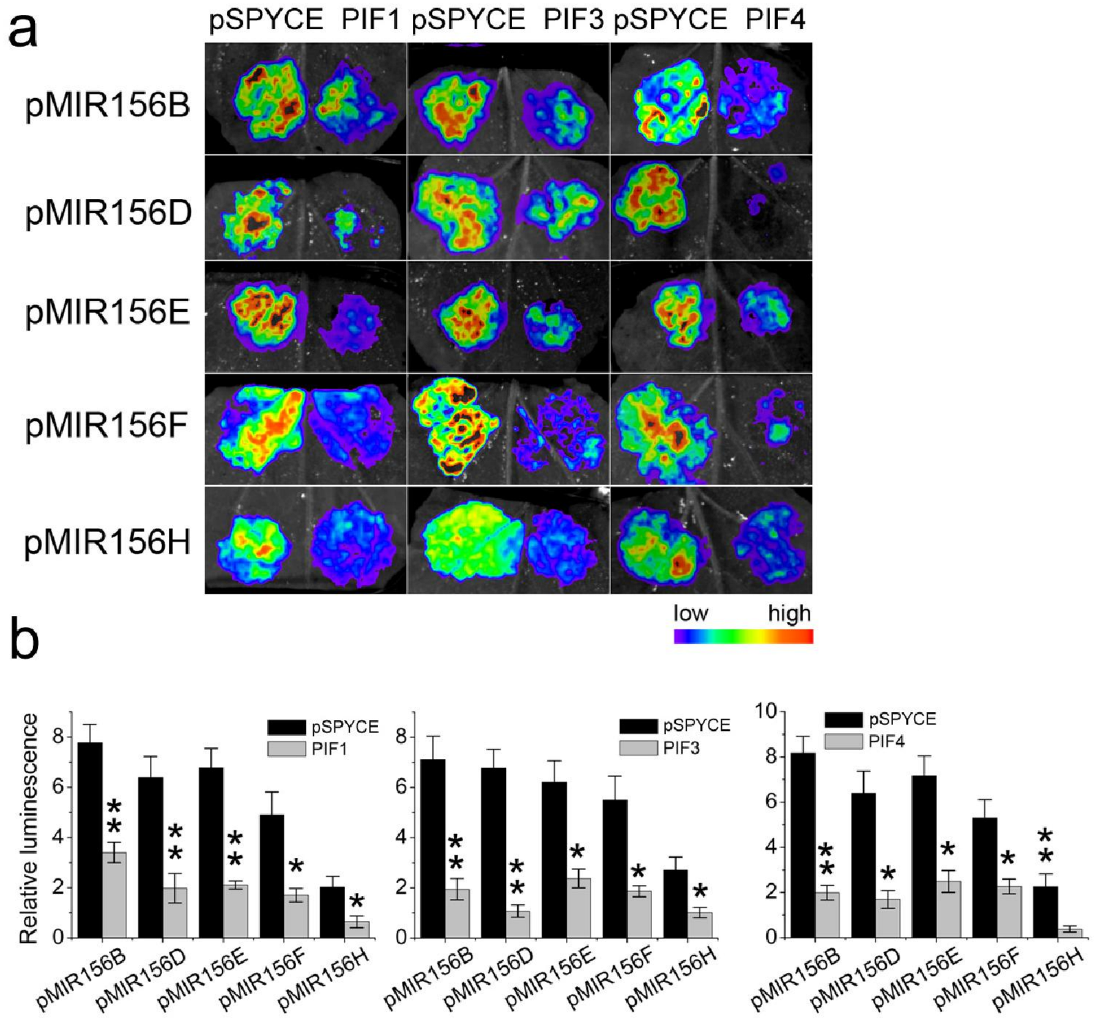

Supplementary Figure 9. PIFs repress the expression of several *MIR156* genes.

(a) PIF1, PIF3 and PIF4 repress the expression of *MIR156B, MIR156D, MIR156E, MIR156F and MIR156H* in transient expression assay. Representative images of *N. benthamiana* leaves 72 hours after infiltration are shown. (b) Quantitative analysis of luminescence intensity in (A). Five independent determinations were assessed. Values given are means ± SD (n=5). *P<0.05 and **P<0.01 by a Student's *t* -test.

Supplementary Figure 10

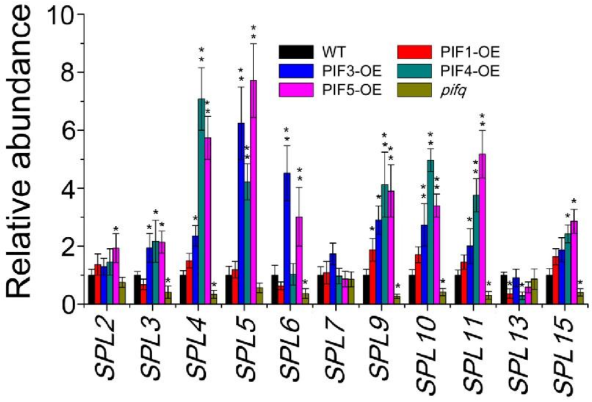

Supplementary Figure 10. The transcript level of several *SPL* genes rise in the *PIF* overexpressors

Eight-day-old wild type, *PIF* overexpressors and *pifq* grown in normal WL conditions were harvested at the same time for RNA extraction and quantitative RT-PCR analyses. Values given represent means ± SD (n=3). *P<0.05 and **P<0.01 by a Student's *t* -test.

Supplementary Figure 11

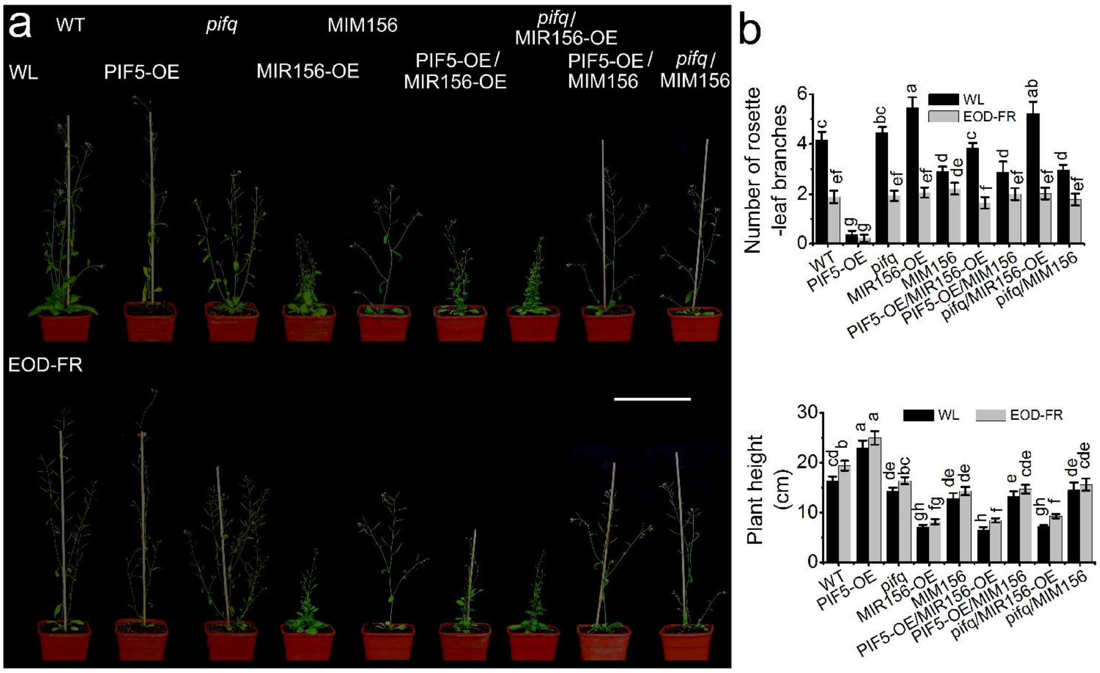

Supplementary Figure 11. ***MIR156s*** **act downstream of** ***PIFs*** **in regulating SAS**

(a) The plant height and rosette-leaf branch number of the adult wild type, *PIF5-OE*, *pifq*, *MIR156-OE*, *MIM156*, and their higher order mutants grown under WL with or without EOD-FR treatment. Eight-day-old seedlings grown under WL were transferred into the soil under WL conditions with or without EOD-FR treatment for four weeks before phenotypic examination. Bar=10 cm. (b) Quantification of the plant height and rosette-leaf branch number of the adult wild type, *PIF5-OE*, *pifq*, *MIR156-OE*, *MIM156*, and their higher order mutants grown under WL with or without EOD-FR treatment. Values given are means ± SD (n=12). Different letters indicate significant differences by two-way ANOVA.

Supplementary Figure 12

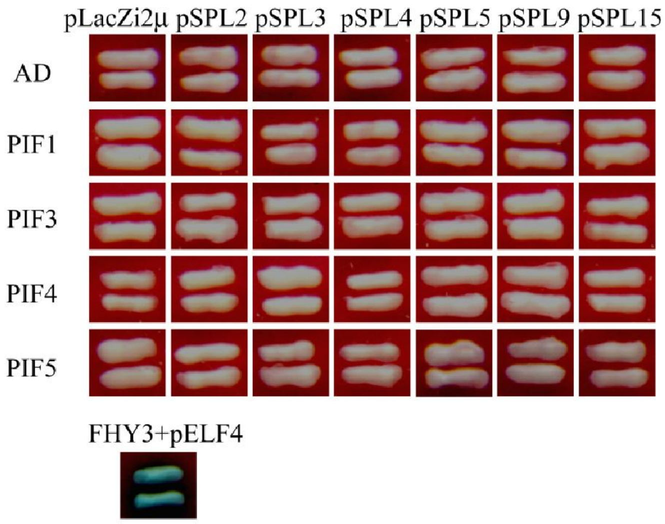

Supplementary Figure 12. No direct binding of PIFs to *SPL* promoters was detected.

Yeast one-hybrid assay showing that there was no direct binding of PIFs to the promoters of *miR156*-targeted *SPLs* genes. The binding of FHY3 to the *ELF4* promoter was used as a positive control (Li et al., 2011).

Supplementary Figure 13

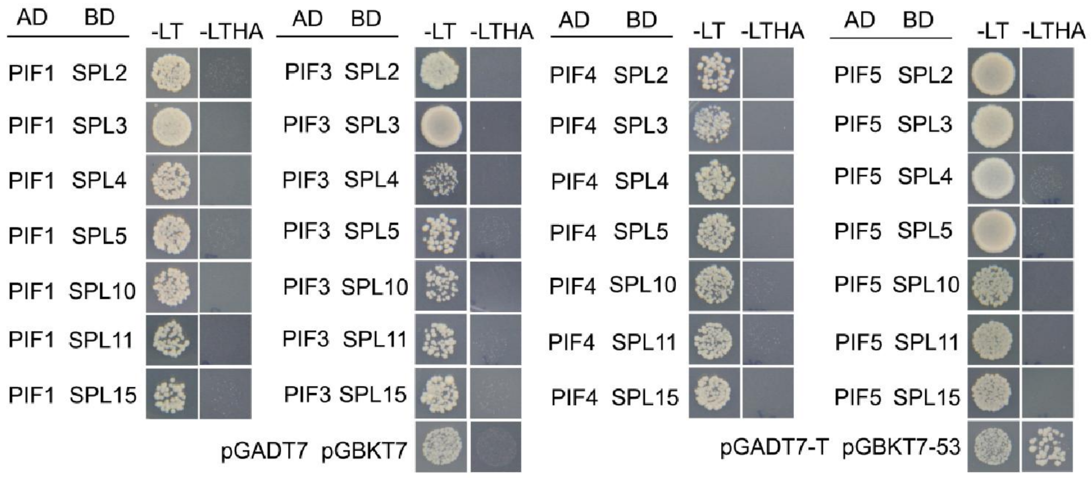

Supplementary Figure 13. No interaction between PIFs and SPLs was detected. Yeast two-hybrid assay showing that there was no interaction between PIFs and SPLs. Seven *miR156*-targeted SPLs were used in this assay. The interaction between pGADT7-T and pGBKT7-53 was used as a positive control.

Supplementary Figure 14

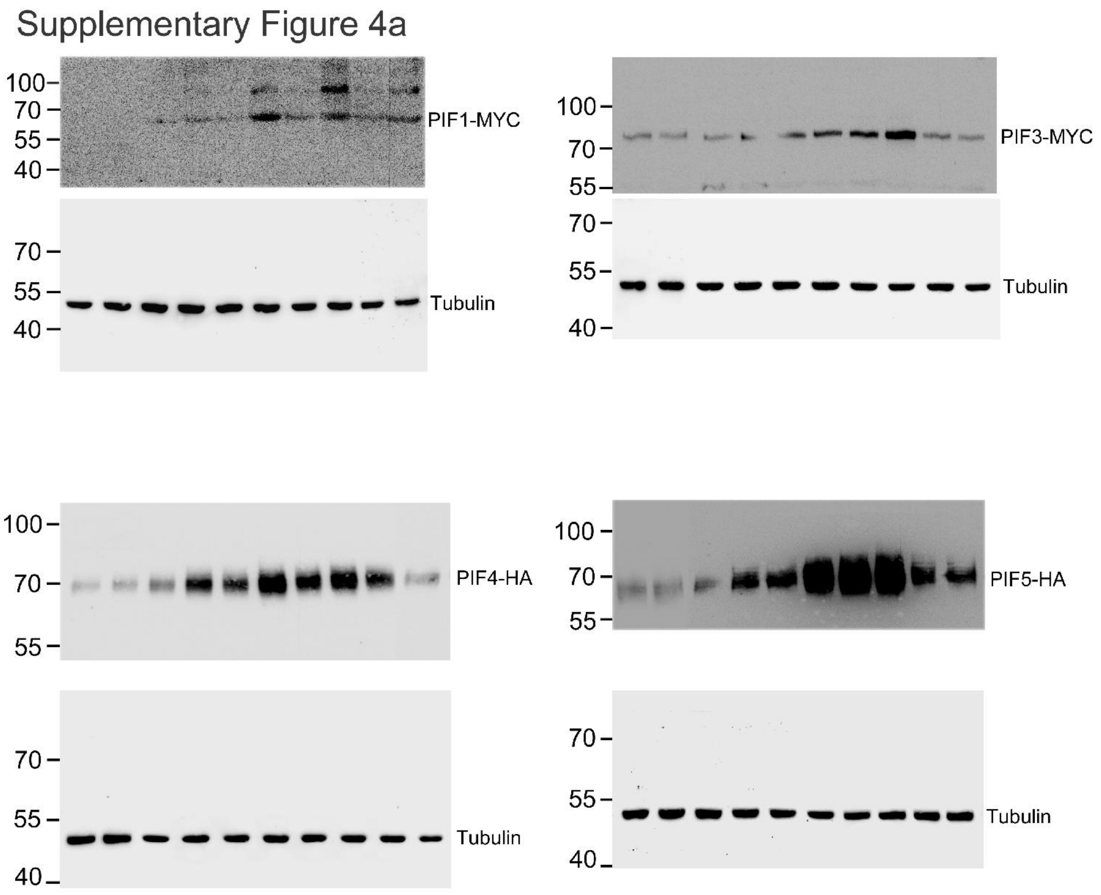

Supplementary Figure 14. The original full images of immunoblots used to prepare figures.

Supplementary Reference

Li, G. *et al*. Coordinated transcriptional regulation underlying the circadian clock in *Arabidopsis*. *Nat Cell Biol.* **13,** 616–622 (2011).
